# Sum of High-Risk Gene Mutation (SHGM): A Novel Attempt to Assist Differential Diagnosis for Adrenocortical Carcinoma with Benign Adenoma, Based on Detection of Mutations of Nine Target Genes

**DOI:** 10.1007/s10528-021-10039-w

**Published:** 2021-02-09

**Authors:** Guo-Yang Zheng, Xue-Bin Zhang, Han-Zhong Li, Yu-Shi Zhang, Jian-Hua Deng, Xing-Cheng Wu

**Affiliations:** Department of Urology, Peking Union Medical College Hospital, Chinese Academy of Medical Sciences and Peking Union Medical College, 1 Shuaifuyuan Road, Dongcheng District, Beijing, 100730 China

**Keywords:** Adrenocortical carcinoma, Adrenocortical adenoma, Differential diagnosis, Gene, Mutation rate

## Abstract

There has been no research on applying gene detection to differential diagnosis of adrenocortical carcinoma (ACC). We attempted to explore a novel auxiliary method for differential diagnosis between ACC with benign adrenocortical adenoma (ACA), based on mutations of target genes in tissues. Nine genes were chosen as target genes, including *TP53, CTNNB1, ARMC5, PRKAR1A, ZNRF3, RB1, APC, MEN1,* and *RPL22*. Exons sequencing of target genes were performed in 98 cases of tissue samples by FastTarget technology, including 41 ACC tissues, 32 ACA tissues, and 25 normal adrenal gland tissues. Significant mutations were detected and identified, and the clinical information was collected, for further comparative analysis and application to assist differential diagnosis of ACC. We identified 132 significant gene mutations and 227 significant mutation sites in 37 ACC tissues, much more than ACA and normal adrenal gland tissues. Mutation rates of 6 genes in ACC tissues were obviously higher than ACA tissues, including *ZNRF3, ARMC5, TP53, APC, RB1,* and *PRKAR1A*, regarded as high-risk genes. The sum of mutated high-risk genes detected in each sample was denominated sum of high-risk gene mutation (SHGM), and the rates of SHGM > 0 and SHGM > 1 in ACC tissues were 73.0% and 62.2%, respectively, both obviously higher than those in ACA tissues, with significant statistic differences. Especially for 8 cases of ACC with diameter < 5 cm, SHGM > 0 and SHGM > 1 were found in 6 samples (75%) and 4 samples (50%), respectively. However, no relevance was found between SHGM and clinical characteristics of ACC. We identified 6 high-risk genes in ACC tissues, with significantly higher mutation rates than ACA or normal adrenal gland tissues. The sum of mutated high-risk genes detected in ACC tissues was denominated SHGM, which was potential to assist the differential diagnosis of ACC with ACA, especially for the small-size ACC.

**Supplementary Information:**

The online version of this article (10.1007/s10528-021-10039-w) contains supplementary material, which is available to authorized users.

## Background

Adrenocortical carcinoma (ACC) is a rare epithelial malignant tumor, derived from adrenal cortical cells with a low annual incidence rate of approximately 0.7–2 people per million (Else et al. 2014). Tumor stage is an important factor affecting the prognosis of ACC patients. Researches showed patients with stage I–II commonly survive for more than 5 years, while the overall survival of ACC patients with stage III was generally 3–5 years. The worst survival prognosis appeared in patients with stage IV, which usually survived merely for less than 1 year (Ayala-Ramirez et al. 2013; Kerkhofs et al. 2013; Tran et al. 2013; Fassnacht et al. 2010). Surgical resection of tumor is recommended as the preferred therapy for ACC, and the completeness of resection is another important influence factor of prognosis of ACC (Grubbs et al. 2010; Crucitti et al. 1996).

In clinical practice, it is sometimes difficult to differentiate ACC from benign adrenocortical adenoma (ACA), especially for small-size tumors, which may lead to inadequate surgical resection and poor survival prognosis. Unfortunately, present pathological examinations, including Weiss system and Ki-67 index, are not infallible for the differential diagnosis of ACC (Wein 2016), so some ACC patients misdiagnosed as ACA before operations might still miss the opportunities of postoperative salvage therapy to improve prognosis.

Therefore, we believe it is still necessary to explore novel differential diagnosis methods for ACC, at least an auxiliary method of current diagnosis system. So far, there has been no genetic method for differential diagnosis of ACC in clinical practice. Mutations of *TP53*, *CTNNB1*, *MEN1,* and *CDKN2A* have been reported associated with ACC, and *PRKAR1A, ZNRF3, RB1, APC,* and *RPL22* were also associated with ACC, based on recent researches (Zheng et al. 2016; Assié et al. 2014). In addition, mutation of *ARMC5* gene was also frequently detected in adrenal disease tissues. Finally, we screened out nine target genes including *TP53, CTNNB1, ARMC5, PRKAR1A, ZNRF3, RB1, APC, MEN1,* and *RPL22*, and detected mutations of target genes in ACC tissues by FastTarget exons sequencing technology. Then we aimed to explore a novel auxiliary method of differential diagnosis for ACC, by comparing and analyzing the mutation characteristics of target genes between ACC to ACA tissues.

## Materials and Methods

### Object

We collected 98 cases of formalin-fixed and paraffin-embedded tissue samples as research subject, including 41 cases of ACC (C1-C41), 32 cases of ACA (A1-A32), and 25 cases of normal adrenal gland tissues (N1-N25), obtained from the patients receiving operations in Peking Union Medical College Hospital (PUMCH) from April 2003 to January 2018. All samples were performed target exons capture and sequencing of 9 target genes by FastTarget technology. All patients were diagnosed by pathological results and clinical follow-up, and two patients (C4 and C19) misdiagnosed as benign ACA by initial pathological examinations were finally diagnosed as ACC until the tumor recurred after surgery. All patients had signed the informed consent forms, and the clinical information was collected, including age, gender, tumor diameter, tumor stage, endocrine function, and Ki-67 index. Tumor diameter was measured by pathological examinations, and tumor stage was based on ENSAT staging criteria. The research was approved by the ethics committee of PUMCH.

### Design

#### Exons Sequencing of 9 Target Genes

Exons capture and enrichment of nine target genes (*TP53, CTNNB1, ARMC5, PRKAR1A, ZNRF3, RB1, APC, MEN1,* and *RPL22*) in all samples were performed by using PCR technology, and Illumina MiSeq Benchtop Sequencer and analysis software were used to detect and screen out the significant mutations in samples. Mutations of target genes were performed further comparative analysis and clinical correlation analysis, to explore auxiliary method for the differential diagnosis of ACC with ACA.

#### Main Instruments and Reagents

Main instruments and reagents involved in this study were demonstrated in Supplementary Table 1.

**Table 1:** Overview of mutations of target genes

	ACC *n* = 37	ACA *n* = 32	Normal gland *n* = 25
Sum of gene mutations	132	30	16
MEN1	20	14	10
ZNRF3	20	2	1
ARMC5	19	2	1
TP53	19	2	2
CTNNB1	13	7	0
APC	12	1	2
RB1	11	0	0
PRKAR1A	9	0	0
RPL22	9	2	0
Number of mutated genes, Median (range)	4.0 (1.5–6.0)	1.0 (0–1.0) *P* < 0.001	1.0 (0–1.0) *P* < 0.001
Sum of mutation sites	227	35	18
Nonsynonymous SNV	169	21	13
Frameshift deletion	19	10	3
Frameshift insertion	0	0	0
Stopgain	19	0	0
Stoploss	1	0	0
Splicing	19	4	2
Number of mutation sites, Medan (range)	4.0 (2.0–10.0)	1.0 (0–2.0) *P* < 0.001	1.0 (0–1.0) *P* < 0.001

#### Sequencing Procedure

Details of exon sequencing procedure are demonstrated in Supplementary Table 2.

**Table 2:** Mutation rates and types of target genes in ACC and ACA tissues

Gene	No. of gene mutation (rate)			Type of mutation (nonsynonymous SNV / Sum)			
	ACC (*n* = 37)	ACA (*n* = 32)	*P*	ACC	ACA		*P*
MEN1	20 (54.1%)	14 (43.8%)	0.472	23/32	10/18	0.352	
ZNRF3	20 (54.1%)	2 (6.3%)	** < 0.001**	19/28	1/2	1.000	
ARMC5	19 (51.4%)	2 (6.3%)	** < 0.001**	36/43	2/2	1.000	
TP53	19 (51.4%)	2 (6.3%)	** < 0.001**	17/25	0/2	0.128	
CTNNB1	13 (35.1%)	7 (21.9%)	0.291	22/26	7/8	1.000	
APC	12 (32.4%)	1 (3.1%)	**0.002**	29/34	1/1	1.000	
RB1	11 (29.7%)	0	**0.001**	12/17	–	–	
PRKAR1A	9 (24.3%)	0	**0.003**	8/13	–	–	
RPL22	9 (24.3%)	2 (6.3%)	0.052	3/9	0/2	1.000	
Total	132	30	–	169/227	21/35	0.075	

#### Bioinformatic Analysis and Mutation Annotation

(a) Original FastQ data file obtained by Illumina high-throughput sequencing platform was performed quality assessment by FastQC software (http://www.bioinformatics. babraham.ac.uk/projects/fastqc), to draw the distribution diagram of base error rate, base quality, and base composition of original data.

(b) All the reads were sequenced twice in forward and reverse (R1 and R2) and were performed sequence calibration by FLASH (Fast-length adjustment of short reads to improve genome assemblies) software (http://www.cbcb.umd.edu/ software/flash), getting the combined FastQ file of R1 and R2. FastX software (http://hannonlab.cshl.edu/fastx_toolkit/index.html) was used to control the quality of sequences covering target regions.

(c) BWA software (http://bio-bwa.sourceforge.net/) was used to compare original sequencing data with human genome data (USCS hg19 version, http://hgdownload.soe.ucsc.edu/goldenPath/hg19/bigZips/), and the comparison results were statistically analyzed by Picard (https://broadinstitute.github.io/picard/).

(d) Preliminary comparison results obtained by BWA software were calibrated by adopting the GATK standard process (https://software.broadinstitute.org/gatk/best-practices/), in order to improve the accuracy of identification of single-nucleotide variants (SNVs) and insertion/deletion (Indel), greatly reducing the false positive and false negative produced in the process of sequencing and comparison.

(e) We used two methods to sequence SNVs and Indel of all samples respectively, which were VarScan (http://varsacan.sourceforge.net/) and GATK Haplotype Caller (https://software.broadinstitute. org/gatk/ best-practices/). We compared the data of detected mutations with latest released exons sequencing data of normal population database, functional database, and disease database, by using ANNOVAR software (http://annovar.openbioinformatics. org/en/latest/). The high-frequency mutations in healthy human and synonymous mutations were removed, in order to discover mutations with biological significance.

### Statistical Analysis

All statistical analysis was conducted by using SPSS software (Statistical Product and Service Solutions version 24.0, USA). The data were described in the form of mean (± standard deviation), median (range), or rate. Results of enumeration data were compared using Chi-squared test and Fisher's exact test. Results of measurement data were compared by nonparametric test. Statistical significance was considered as p ≤ 0.05.

## Results

### Quality Control

We eliminated 4 unqualified samples (C3, C5, C7, C17), and the other 94 samples were qualified for further bioinformatic analysis (Supplementary Fig. 1). Quality analysis showed mean sequencing depth of all samples was 733X (Supplementary Fig. 2), and sequencing depth of target gene fragments was above 10X in 95.1% samples (Supplementary Fig. 3). In conclusion, results of quality analysis indicated that the results of sequencing were reliable.

**Fig. 1:**
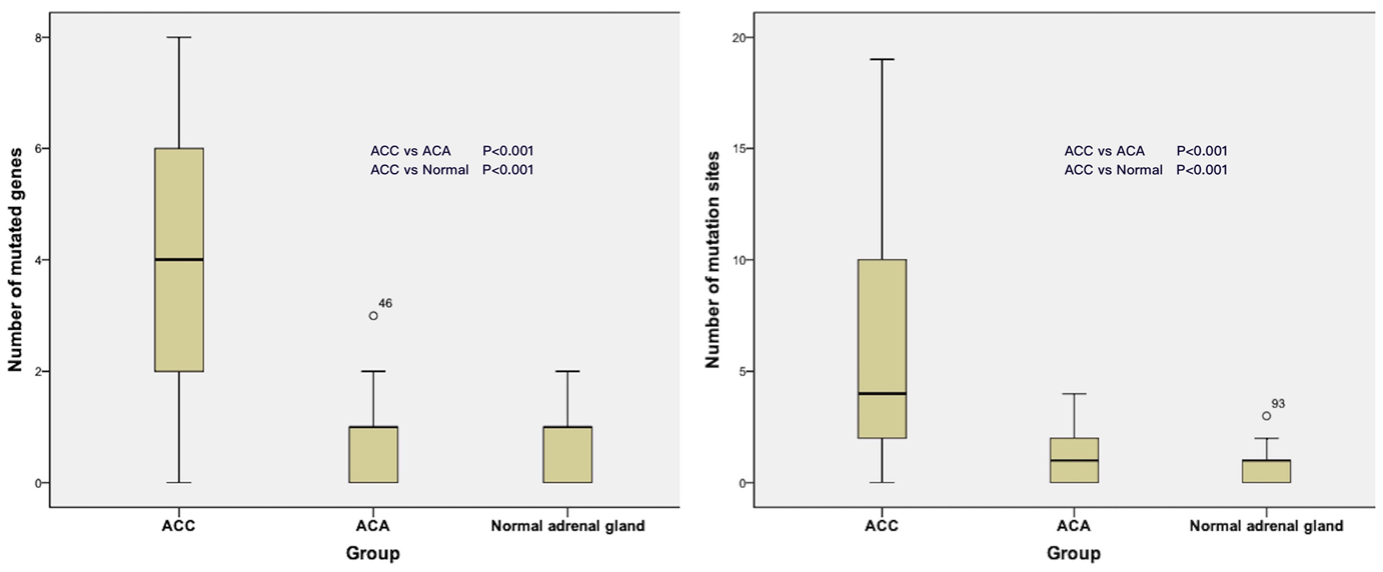
Kruskal–Wallis test box plot of mutated target genes and mutation sites in 3 groups of tissues

**Fig. 2:**
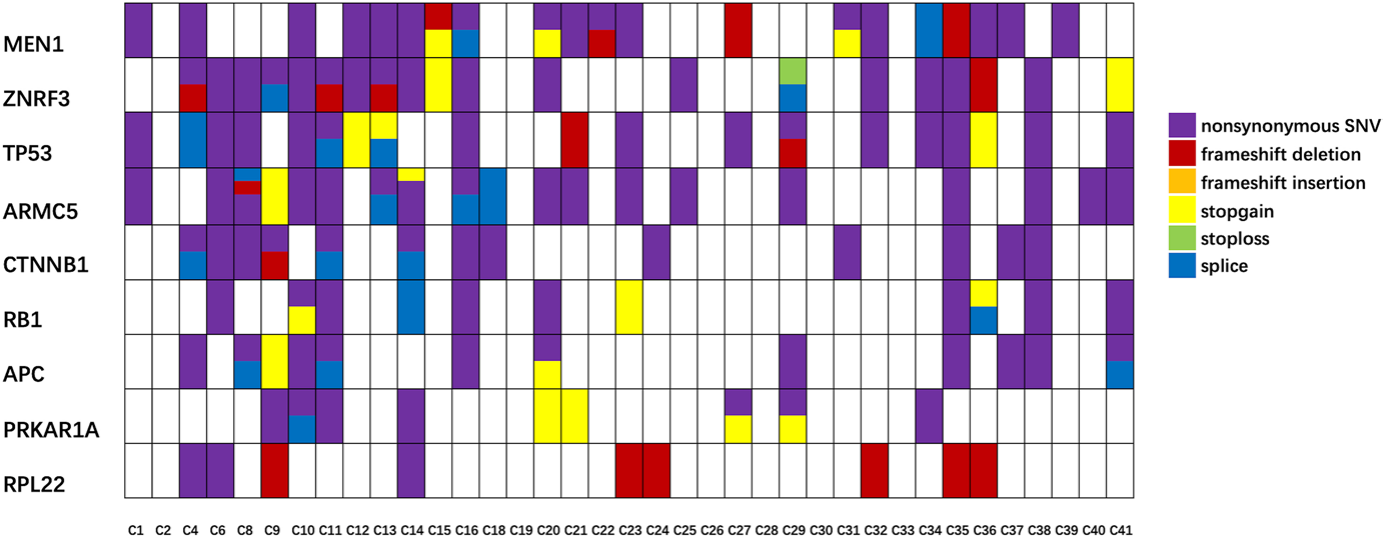
The mutation information of nine target genes in ACC tissues. The labels of samples were sample IDs

**Fig. 3:**
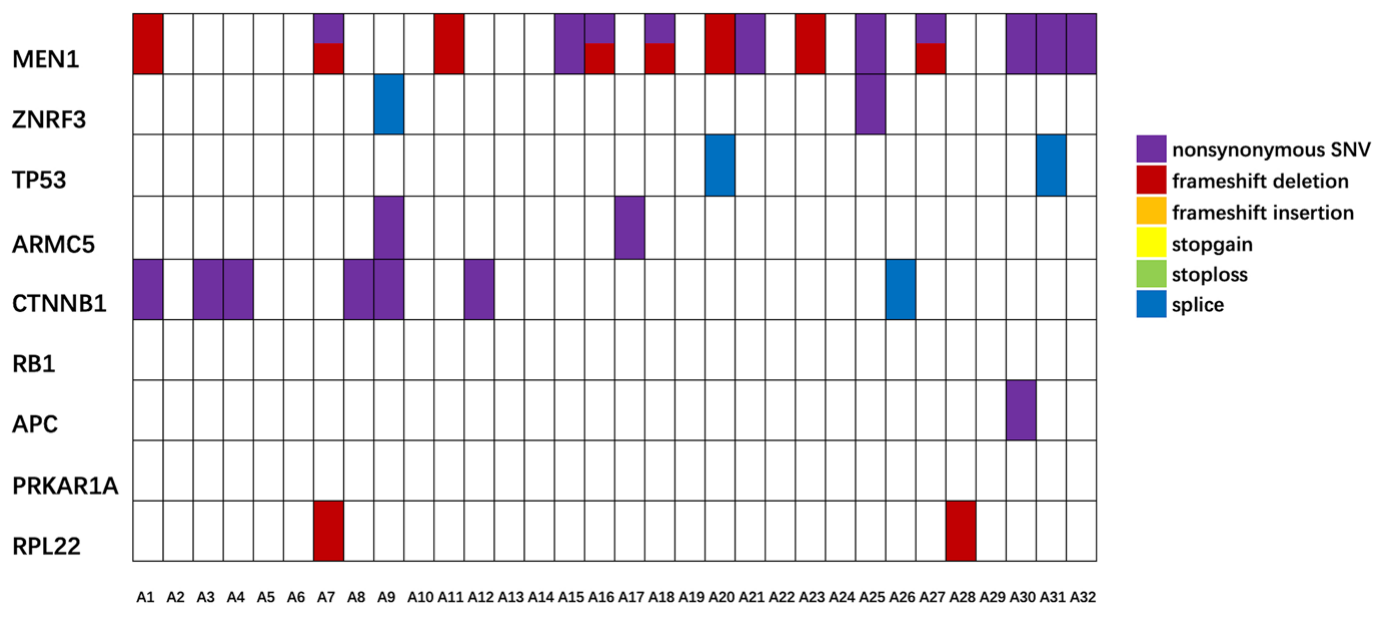
The mutation information of nine target genes in ACA tissues. The labels of samples were sample IDs

### Overview of Sequencing Results of Target Gene Mutations

We sequenced and analyzed mutation information of 9 target genes in 94 tissue samples, including 37 ACC, 32 ACA, and 25 normal adrenal gland tissue samples, using FastTarget technology to perform target exons sequencing. Exons capture and sequencing of target genes, which were *TP53, CTNNB1, ARMC5, PRKAR1A, ZNRF3, RB1, APC, MEN1,* and *RPL22*, included 202 PCR sequencing fragments in all.

We used VerScan and GATK HaplotypeCaller two methods to sequence single-nucleotide variants (SNVs) and insertion/ deletion (Indel) of all samples, respectively, compared with normal population database as mentioned in Design section. Totally, we detected 3017 exonic mutations, 3732 intronic mutations, 458 splicing mutations, 317 5′-UTR (untranslated region) mutations, and 39 3′-UTR mutations in 94 tissue samples. Focusing solely on exonic mutations, we detected 1062 nonsynonymous SNVs, 1830 synonymous SNVs, 27 stopgain mutations, 1 stoploss mutation, 95 frameshift deletion, and 1 nonframeshift insertion mutation, without nonframeshift deletion or frameshift insertion was detected. Because some mutations appeared repeatedly in samples, we finally identified 505 kinds of exonic mutations and 50 kinds of splicing mutations. Then, we targeted the nonsynonymous SNVs, stopgain, stoploss, frameshift deletion, and splicing mutations as analysis subjects.

Due to the lack of normal tissues in same patients for comparative analysis, we performed comparative analysis of all detected mutations by using ANNOVAR. Then we removed normal variations, high-frequency mutations in healthy human, and synonymous mutations, to discover mutations with biological significance. Finally, we identified that 178 gene mutations with biological significance occurred in the 94 tissue samples, with 280 significant mutation sites.

In 37 ACC tissue samples, 132 significant gene mutations and 227 significant mutation sites were identified, including 169 nonsynonymous SNVs, 19 stopgain mutations, 1 stoploss mutation, 19 frameshift deletions, and 19 splicing mutations. The median numbers of target gene mutations and mutation sites occurring in ACC samples were 4.0 (1.5–6.0) and 4.0 (2.0–10.0), respectively. The maximum of gene mutations was 8, detected in sample C35, and the maximum of mutation sites was 19, detected in sample C11. In 32 samples of ACA group, median numbers of gene mutations and mutation sites were 1.0 (0–1.0) and 1.0 (0–2.0), respectively. And the counterparts in 25 normal adrenal gland tissues were both 1.0 (0–1.0) (Table 1). The statistical results showed that the amount of target gene mutations and mutation sites detected in ACC tissues were both higher than those in ACA and normal adrenal gland tissues, with significant difference (P < 0.001) (Fig. 1). The results indicated that exons sequencing of target genes in this research may be potential to assist the distinguish of ACC.

### Comparative Analysis of Target Gene Mutations in ACC

Although in 6 samples (C2, C19, C26, C28, C30, and C33) we failed to detect any significant gene mutations, 132 significant gene mutations and 227 mutation sites were identified in other 31 ACC samples. The mutation rates of the nine target genes in ACC tissue samples from high to low were 54.1% (20/37) of *MEN1*, 54.1% (20/37) of *ZNRF3*, 51.4% (19/37) of *ARMC5*, 51.4% (19/37) of *TP53*, 35.1% (13/37) of *CTNNB1*, 32.4% (12/37) of *APC*, 29.7% (11/37) of *RB1*, 24.3% (9/37) of *PRKAR1A*, and 24.3% (9/37) of *RPL22*. In the 227 mutation sites, the incidences of mutation types were: 74.4% (169/227) of nonsynonymous SNV, 8.4% (19/227) of stopgain mutation, 8.4% (19/227) of frameshift deletions, 8.4% (19/227) of splicing mutation, and 0.4% (1/227) of stoploss mutation. The main mutation type of *RPL22* gene occurring in ACC tissues was frameshift deletion (66.7%, 6/9), while the main mutation type of the other genes was nonsynonymous SNV. The mutation information of nine target genes in ACC is shown in Fig. 2.

**Fig. 4:**
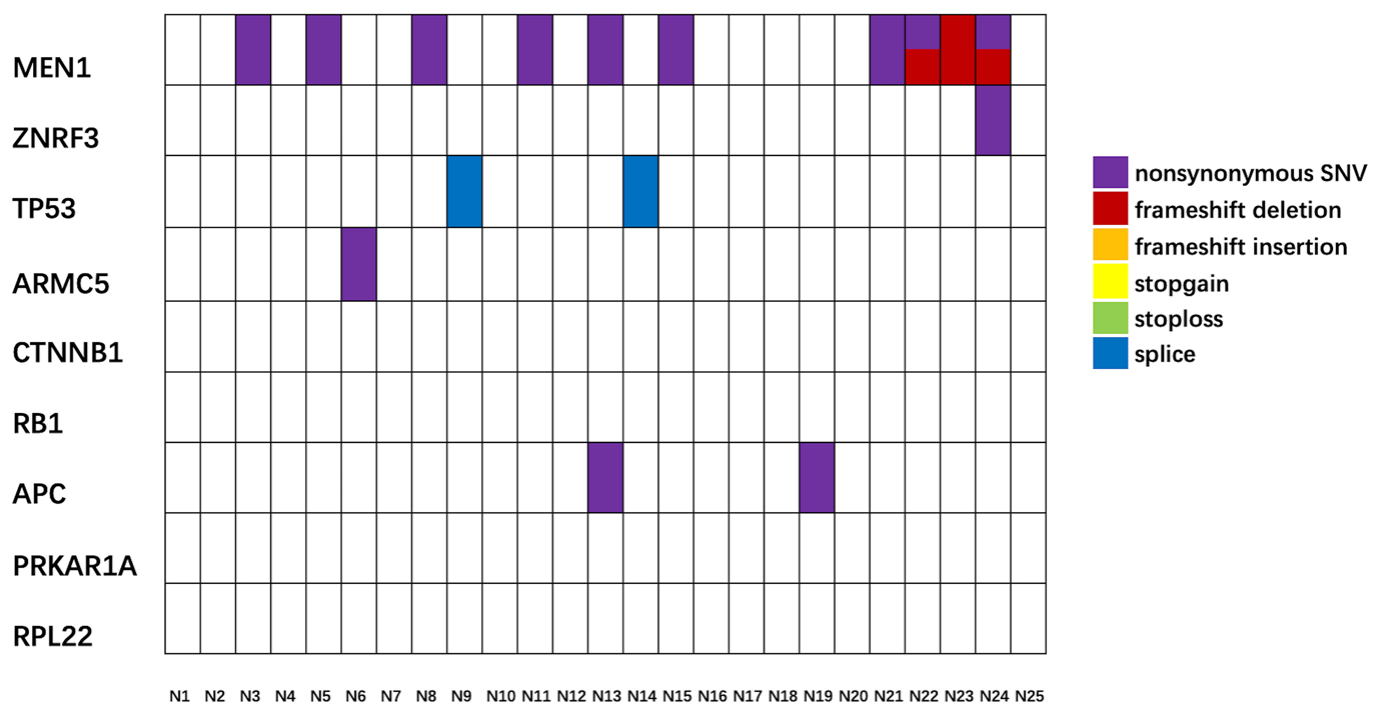
The mutation information of nine target genes in normal adrenal gland tissues. The labels of samples were sample IDs

Comparative analysis of mutations of target genes between ACC tissues to ACA tissues demonstrated that mutation rates of 6 genes in ACC group were obviously higher than those in ACA group, including *ZNRF3, ARMC5, TP53, APC, RB1*, and *PRKAR1A*. As for the other 3 genes (*MEN1, CTNNB1*, and *RPL22*), there was no difference in mutation rate or mutation type (Table 2). The above results suggested that mutations of *ZNRF3, TP53, ARMC5, APC, RB1,* and *PRKAR1A* may be potential to assist the differential diagnosis between ACC and ACA. Comparative analysis between ACC and normal adrenal gland showed that mutation rates of 7 genes in ACC tissues were higher than those in normal tissues, which were *ZNRF3, ARMC5, TP53, CTNNB1, APC, RB1,* and *PRKAR1A* (Table 3). Mutation information of nine target genes in ACC, ACA, and normal adrenal gland tissues is demonstrated in Fig. 2, Fig. 3, and Fig. 4, respectively.

**Table 3:** Mutation rates and types of target genes in ACC and normal tissues

Gene	No. of gene mutation (rate)			Type of mutation nonsynonymous SNV/Sum		
	ACC (*n* = 37)	Normal (*n* = 25)	* P*	ACC	Normal	*P*
MEN1	20 (54.1%)	10 (40.0%)	0.311	23/32	9/12	1.000
ZNRF3	20 (54.1%)	1 (4.0%)	** < 0.001**	19/28	1/1	1.000
ARMC5	19 (51.4%)	1 (4.0%)	** < 0.001**	36/43	1/1	1.000
TP53	19 (51.4%)	2 (8.0%)	** < 0.001**	17/25	0/2	0.128
CTNNB1	13 (35.1%)	0	**0.001**	22/26	–	–
APC	12 (32.4%)	2 (8.0%)	**0.031**	29/34	2/2	1.000
RB1	11 (29.7%)	0	**0.002**	12/17	–	–
PRKAR1A	9 (24.3%)	0	**0.003**	8/13	–	–
RPL22	9 (24.3%)	0	0.052	3/9	–	–
Total	132	16	–	169/227	13/18	0.835

### SHGM to Assist Differential Diagnosis of ACC with ACA

We proposed a novel concept that was denominated “sum of high-risk gene mutation,” abbreviated to SHGM, as an auxiliary method for differential diagnosis of ACC with ACA, by exons capture and sequencing of target genes. We elected six genes as high-risk mutant genes related to ACC, which were *ZNRF3, TP53, ARMC5, APC, RB1,* and *PRKAR1A,* based on above analysis results. Mutation rates of high-risk genes in ACC tissues were much higher than those in ACA tissues, and we count the sum of mutated high-risk genes detected in each sample, regarded as the SHGM of the sample.

Then, we calculated the SHGM of every samples in ACC group and ACA group. For the 37 samples of ACC tissues, SHGM > 0 was found in 27 samples (73.0%), and SHGM > 1 was identified in 23 samples (62.2%). However, we found SHGM > 0 in only 6 samples (18.8%) of ACA group, and there was only 1 sample (A9) with SHGM > 1 (3.1%). The rates of SHGM > 0 and SHGM > 1 in ACC tissues were both obviously higher than those in ACA tissues, with significant statistic differences (Table 4). More importantly, in the 8 samples of ACC with diameter less than 5 cm, which tended to be misdiagnosed as ACA, we found SHGM > 0 in 6 samples (75%) and SHGM > 1 in 4 samples (50%) (Table 5). It indicated that the SHGM might contribute to differential diagnosis between ACC to ACA in some degree, especially for the ACC with small diameter. If we set SHGM > 0 as reference standard to detect the efficacy of differential diagnosis between ACC with ACA tissue samples included in our research, the specificity and sensibility of identification of ACC were 72.2% and 81.8%, respectively. If the reference standard was set as SHGM > 1, the specificity and sensibility were 68.9% and 95.8%, respectively.

**Table 4:** Sum of high-risk gene mutation (SHGM) in ACC vs ACA

	ACC Group	ACA Group	*P*
SHGM, median (range)	2 (2–4)	0 (0–0)	< 0.001
Rate of SHIRMS > 0	73.0% (27/37)	18.8% (6/32)	< 0.001
Rate of SHIRMS > 1	62.2% (23/37)	3.1% (1/32)	< 0.001

**Table 5:** Sum of high-risk gene mutation (SHGM) in ACC with diameter ≤ 5 cm

Sample Number	C1	C4	C11	C19	C22	C29	C37	C40
Diameter, cm	5.0	3.8	4.5	4.0	3.0	2.0	4.7	4.5
SHGM ZNRF3 TP53 ARMC5 APC RB1 PRKAR1A	2 − ** + ** ** + ** − − −	3 ** + ** ** + ** − ** + ** − −	6 ** + ** ** + ** ** + ** ** + ** ** + ** ** + **	0 − − − − − −	0 − − − − − −	5 ** + ** ** + ** ** + ** ** + ** − ** + **	1 − − − ** + ** − −	1 − − ** + ** − − −

We also performed correlation analysis between SHGM and clinical characteristics of ACC, including gender, age, endocrine function, diameter, tumor stage, and Ki-67 index. The continuous variable such as age, diameter, and Ki-67 index, was compared with SHGM by nonparametric test, while the counting variable, such as gender, endocrine function, and tumor stage, was compared with SHGM by Chi-squared test or Fisher's exact test. Unfortunately, we did not find any statistical relevance or significant difference between SHGM and above characteristics (Table 6), which indicated SHGM might be incapable of predicting clinical characteristics or prognosis of ACC.

**Table 6:** Correlation analysis between SHGM with clinical characteristics in ACC

Clinical characteristics	Result	R-value	*P*
Age, years, mean ± SD (95% CI)	44.3 ± 12.9( 39.8–48.8)	0.025	0.888
Diameter, cm, mean ± SD (95% CI)	8.5 ± 3.5( 7.2–9.7)	0.210	0.213
Ki-67 index (%), median (range)	20 (10–25)	0.097	0.593

Clinical characteristics	*n* (%)	SHGM	*P*
Sex Male Female	12 (32.4%) 25 (67.6%)	3.5 (0.25–4.75) 2 (0–3.5)	0.419
Endocrine function Nonfunction Function	17 (45.9%) 20 (54.1%)	3 (0–5) 2 (1–3.5)	0.631
Tumor stage Stage I–II Stage III–IV	19 (51.4%) 18 (48.6%)	2 (0–4) 3 (0.75–5)	0.240

## Discussion

### Importance and Limitation of Pathological Differential Diagnosis of ACC

Adrenocortical carcinoma is a rare malignant tumor with a low incidence and relatively poor prognosis (Else et al. 2014; Fassnacht et al. 2011). Complete resection of tumor and surrounding tissues is considered as the optimal treatment of ACC, and the completeness of surgical resection is an important influencing factor on the prognosis of ACC (Bilimoria et al. 2008). Complete resection range should include at least the whole tumor with complete capsule, the whole adrenal gland, and as much surrounding adipose tissue as possible. Research has shown that postoperative adjuvant therapy, such as radiotherapy or mitotane treatment, might contribute to improving the postoperative prognosis of ACC, especially for those tumors resected with positive margin (Grubbs et al. 2010; Terzolo et al. 2007). Unfortunately, some small-size ACC might be misdiagnosed as benign ACA, which may mislead surgeons and lead to incomplete tumor resection scope. Therefore, accurate differential diagnosis is important for ACC patients to choose appropriate therapy strategy. Pathological diagnosis is the most important defining characteristics for differential diagnosis of ACC, which could help deciding the requirement of postoperative adjuvant therapy and guiding the follow-up strategy. More importantly, for the ACC patients who were misdiagnosed as benign ACA and received incomplete tumor resection, accurate postoperative pathological diagnosis could warn the patients to perform rescue therapy as soon as possible, such as secondary surgery, mitotane therapy, or radiotherapy, in order to improve the prognosis of patients.

However, in clinical practice, pathological diagnosis is not entirely reliable to distinguish ACC from ACA, especially for the small-size tumors with diameter less than 5 cm. Cytological characteristics of ACC tissues are similar with ACA tissues under the microscope sometimes, due to the endocrine function of adrenocortical tumors (Wein 2016). Cells of benign adenoma with endocrine function may appear cytological characteristics similar to malignant ACC, such as caryogram, pathological mitosis, and cell ultrastructure including the change of mitochondria, basement membrane, or endoplasmic reticulum. So far, there have been no particular specific immunohistochemical markers for accurate identification of ACC. Current reference immunohistochemical markers clinically used for differential diagnosis of ACC, merely could indicate whether the tumor derived from adrenal cortex or not (Duregon et al. 2013; Ghorab et al. 2003; Jorda et al. 2002; Arola et al. 2000). Those markers, such as α-inhibin, synaptophysin, melanA, calretinin, SF1, and so on, were mainly used to distinguish adrenocortical tumors from adrenal pheochromocytomas, adrenal metastases, or other types of retroperitoneal neoplasms, while those marker could not be regarded as specific markers for ACC.

Weiss system is regarded as the most effective and widely used clinical diagnostic system for ACC at present. In the Weiss system, there are 9 criteria in total, and the presence of 3 or more criteria will suggest the diagnosis of ACC, with sensitivity and specificity both more than 90% (Aubert et al. 2002). Ki-67 index is another important diagnostic criterion for differential diagnosis of ACC (Libé 2015). It is generally considered that Ki-67 index more than 5% is probably associated with ACC, and the higher index will indicate the worse prognosis, such as overall survival and progression free survival (Morimoto et al. 2008). In addition, destruction of intracellular reticular fibers observed by cytoskeleton staining could also be regarded as a differential criterion for ACC (Papotti et al. 2011; Mihai 2015). Unfortunately, differential diagnosis of ACC only based on the above pathological methods may be not entirely reliable. In clinical practice, a few patients pathologically diagnosed with ACA finally proved to be malignant ACC and might miss the opportunity of radical treatment or rescue therapy. Similarly, a few patients pathologically diagnosed with ACC finally did not appear as malignant biological properties.

Therefore, it is still necessary and valuable to explore another novel identification method to assist the differential diagnosis of ACC. In consideration of the current limitation of pathological diagnostic methods and the absence of genetic diagnostic methods, we aimed to explore a novel differential diagnosis system based on the condition of gene mutations in ACC tissues. We expected to improve the accuracy of differential diagnosis for ACC and contribute to improve the prognosis of ACC patients who were misdiagnosed as benign ACA, by using this novel genetic diagnostic method as supplement or combination of current diagnosis methods such as the Weiss system and Ki-67 index. Then, the ACC patients misdiagnosed as benign tumor before operation could receive timely salvage therapy in order to improve survival prognosis.

### Current Status of Genetic Researches of Adrenocortical Carcinoma

Some past researches have demonstrated that mutations of *TP53* gene, *CTNNB1* gene, *MEN1* gene, and *CDKN2A* gene might be associated with ACC (Juhlin et al. 2015; Gaujoux et al. 2008; Tissier et al. 2005; Barzon et al. 2001; Kjellman et al. 1999; Nobori et al. 1994). There were two convincing researches published on prestigious journals recently, which provided more reliable data of genetic researches in ACC (Zheng et al. 2016; Assié et al. 2014). A research published in Nature Genetics performed whole exons sequencing on 45 cases of ACC tissues (Assié et al. 2014). The research identified several mutant genes associated with ACC, such as *ZNRF3, CTNNB1, TP53, CDKN2A, RB1,* and *MEN1*. In addition, it discovered that copy number variations of some genes were also related with ACC, such as amplification of *TERT* gene (5p.15.33), and homozygous deletion of *CDKN2A* (9p21.3), *RB1* (13q14), and *ZNRF3* (22q12.1). Another famous research published in Journal Cell in 2016, detected totally 8814 somatic SNVs and small Indels, including 6664 nonsynonymous mutations and 2150 synonymous mutations (Zheng et al. 2016). This study identified several significant mutant genes in ACC tissues, including *TP53, CTNNB1, MEN1, PRKAR1A,* and *RPL22*. Increased copy numbers of *TERT* gene (5p15.33), *TERF2* gene (16q22.1), *CDK4* gene (12q14.1), and *CCNE1* gene (19q12), as well as deletions of *RB1* gene (13q14.2), *CDKN2A* gene (9p21.2), and *ZNRF3* gene (22q12.1), were also detected in ACC tissues.

According to above researches (Zheng et al. 2016; Assié et al. 2014), *ZNRF3* gene mutation was newly discovered associated with ACC, with a mutation rate of approximately 21% according to above researches. *ZNRF3* gene encodes transmembrane E3 ubiquitin ligase on cell surface, which could inhibit function of Wnt/β-catenin signaling pathway, so mutation of *ZNRF3* could result in the over-activation of this signaling pathway. Besides, mutations of *CTNNB1, MEN1,* and *APC* also could cause over-activation of Wnt/β-catenin pathway, and mutation rates of above genes detected in ACC were approximately 20%, 7%, and 3%, respectively (Zheng et al. 2016; Assié et al. 2014). The Wnt/β-catenin signaling pathway was involved in regulating cell adhesion, proliferation, apoptosis, and other cellular functions, which was usually found over-activated in ACC tissues (Zheng et al. 2016). *TP53/Rb* was another signaling pathway associated with ACC, which could be influenced by mutation of gene *TP53* and *Rb1* (Zheng et al. 2016). *TP53* was located in chromosome 17 p13, involved in cell proliferation and apoptosis, and the mutation rate of *TP53* detected in ACC was reported as 21.3%. In addition, *PRKAR1A, RPL22,* and *NF1* were newly discovered genes related to ACC recently, and mutation rates detected in ACC were 8%, 7%, and 5%, respectively (Zheng et al. 2016; Assié et al. 2014). The mutation of *ARMC5* gene was frequently detected in primary macronodular adrenal hyperplasia, but there has been no research detecting mutation status of ARMC5 in ACC tissues (Zhang et al. 2018).

According to the above research data, we finally selected 9 genes associated with ACC as target genes, which were *ZNRF3, TP53, CTNNB1, PRKAR1A, MEN1, RB1, APC, RPL22,* and *ARMC5*, in order to explore mutation characteristics and clinical value of the target genes in ACC tissues. Then, we expected to apply it to the auxiliary differential diagnosis of ACC after operations, in order to improve the prognosis of preoperative misdiagnosed ACC patients, by timely salvage therapy such as secondary surgery, radiotherapy, and mitotane treatment.

### Clinical Significance and Limitations of SHGM

In this research, we analyzed the mutation rates and types of 9 target genes in ACC tissues by exons sequencing and compared the data with that of ACA and normal adrenal gland tissues. The results showed that the mutation rates of 6 genes detected in ACC tissues were significantly higher than those in ACA and normal adrenal gland tissues, which were *ZNRF3, TP53, ARMC5, APC, RB1,* and *PRKAR1A*. We regarded the 6 genes as high-risk genes associated with ACC and proposed a novel concept named sum of high-risk mutation genes (SHGM), defined as the count of mutations of above 6 high-risk genes detected in ACC tissues. We applied SHGM to the auxiliary differential diagnosis of ACC, which is the innovation of this research. In the 37 cases of ACC tissues, SHGM > 0 was identified in 27 samples (73.0%), and SHGM > 1 was identified in 23 samples (62.2%). While in the 32 cases of ACA tissues, there were only 6 samples with SHGM > 0 (18.8%) and only one sample with SHGM > 1 (3.1%), and the difference with ACC group was statistically significant (P < 0.05). The results indicated that the SHGM may be helpful in the differential diagnosis of adrenocortical carcinoma and benign adenoma. If we set SHGM > 1 as reference index for diagnosis of ACC, the sensibility for identification of ACC could be up to 95.8%. However, the specificity was only 68.9%, which indicated that some benign ACA patients might be overestimated as malignant ACC, leading to the possibility of over-treatment. Therefore, we expected SHGM to be an auxiliary diagnostic index, which should be applied to the differential diagnosis of ACC together with clinical existing diagnostic methods, such as the Weiss system, Ki-67 index, and cellular reticular fiber structure staining, in order to maximize the diagnostic accuracy.

More importantly, we found that SHGM might be more helpful and valuable in the differential diagnosis of small-size ACC with benign adenoma, reflecting the clinical significance and innovation of the establishment of SHGM. In the 8 cases of ACC with tumor diameters ≤ 5 cm, SHGM > 0 was identified in 6 cases (75%), and the rate of SHGM > 1 was 50%. Among the 8 cases, there were two patients (C4 and C19) misdiagnosed as benign ACA by postoperative pathological diagnosis. Unfortunately, the tumors recurred rapidly in the two patients due to the absence of timely salvage treatment after operations, with DFS of 9.6 months for C4 and 16.0 months for C19, respectively, while researches showed the survival of small-size adrenocortical carcinoma were usually more than 5 years (Kerkhofs et al. 2013), much better than the surviving for approximately 3 years of the above 2 patients. The SHGM detected in C4 and C19 were 3 and 0, respectively, so the application of SHGM for differential diagnosis could have improved the prognosis of C4 patient by timely salvage therapy after operation. Therefore, we considered that the SHGM was helpful for the auxiliary differential diagnosis of ACC after surgery and valuable to improve the clinical prognosis of ACC patients, especially for the small-size tumors with diameters ≤ 5 cm, which might be tended to be misdiagnosed as benign tumor.

It is inevitable that there are some limitations in this research. First, the sequencing data of tumor tissue lacked the control of normal tissue of the same patient, because the ACC samples were obtained from formalin-fixed and paraffin-embedded tissues of patients who had received operations in our hospital in the past. Most of the patients have died or lost contact, so we were unable to obtain the normal tissues from the same patients as control to screen significant mutant genes and mutation sites. Then, we compared the sequencing results with the present data of whole exon and genome-sequencing results in normal population, which might be one of the reasons why the mutation rates of the target genes in our research were a little higher than those of the corresponding genes published in past studies, and the other possible reasons might include racial differences and the differences of sequencing platforms or sequencing technology. All tissue samples in our study were compared with the same database, and the results of quality control showed that the amplifications of target genes fragments were qualified. We believed that the results of our research were reliable and clinically significant, since the original intention was expecting to apply SHGM to auxiliary differential diagnosis of ACC with ACA, rather than exploring the pathogenic mutant genes and mutation sites of adrenocortical carcinoma.

Second, the SHGM system only including 6 target genes based on the sequencing and analysis results in this study, and we believe that there are still some genes with differential diagnosis significance for ACC to be found and studied. Mutations occurring in other ACC relevant genes and noncoding regions may also be important, and increasing the scope of candidate genes and sequence regions may be helpful for increasing the power of diagnosis. At last, we failed to find any significant statistic correlations between SHGM with other clinical characteristics of ACC, including age, gender, tumor diameter, tumor stage, endocrine function, and Ki-67 index, so we do not recommend the SHGM for the evaluation of clinical features and prognosis of ACC. Comparing tissues collected from the same individual, improved techniques in computational modeling, and statistical analysis would be helpful for developing this diagnosis method.

## Conclusions

In this research, we performed target exons sequencing of 9 target genes in ACC, ACA, and normal adrenal gland tissues. Analysis results identified 6 high-risk genes associated with ACC, of which the mutation rates in ACC tissues were significantly higher than those in ACA or normal adrenal gland tissues, including *ZNRF3, TP53, ARMC5, APC, RB1,* and *PRKAR1A*. We proposed the concept of SHGM defined as the sum of mutated high-risk genes detected in ACC tissues, and the rates of SHGM > 0 and SHGM > 1 in ACC were significantly higher than those in ACA tissues, which suggest SHGM might be potential to assist the differential diagnosis of ACC with ACA, especially for ACC with small tumor size.

## Supplementary Information

Multiple PCR results of 94 cases of eligible samples for quality control. A1 for negative control, A2 for positive control, M for DL2000Marker, other symbols of B, C, D, E, F, G, H, and A3-A12 for all the 94 cases of samples. A-H were well positions, not sample IDs (jpg 4130 kb)

Sequencing depth of all 94 samples for quality analysis. Ranged from 190X to 1536X, and the mean was 733X. The labels of samples were sample IDs (jpg 4485 kb)

Sequencing depth of target gene fragments was above 10X in 95.1% samples. The labels of samples were sample IDs (jpg 6262 kb)

Electronic supplementary material 4 (DOCX 60 kb)

Electronic supplementary material 5 (DOCX 90 kb)

## Data Availability

All the data were collected and analyzed by medical team of Han-Zhong Li in PUMCH. Editors and reviewers could send e-mail to hzlipumch@163.com to require any data associated with the study.

## Abbreviations

SHGM: Sum of high-risk gene mutation
ACC: Adrenocortical carcinoma
ACA: Adrenocortical adenoma
PUMCH: Peking Union Medical College Hospital
ENSAT: European Network for the Study of Adrenal Tumors
PCR: Polymerase chain reaction
FLASH: Fast-length adjustment of short reads to improve genome assemblies
SNVs: Single-nucleotide variants
Indel: Insertion/ deletion
UTR: Untranslated region
