# Supplementary figures and images for: Sum of High-Risk Gene Mutation (SHGM): A Novel Attempt to Assist Differential Diagnosis for Adrenocortical Carcinoma with Benign Adenoma, Based on Detection of Mutations of Nine Target Genes
Source: Biochem Genet. 2021 Feb 9;59(4):902–18. doi: 10.1007/s10528-021-10039-w (PMC8249247; doi:10.1007/s10528-021-10039-w)

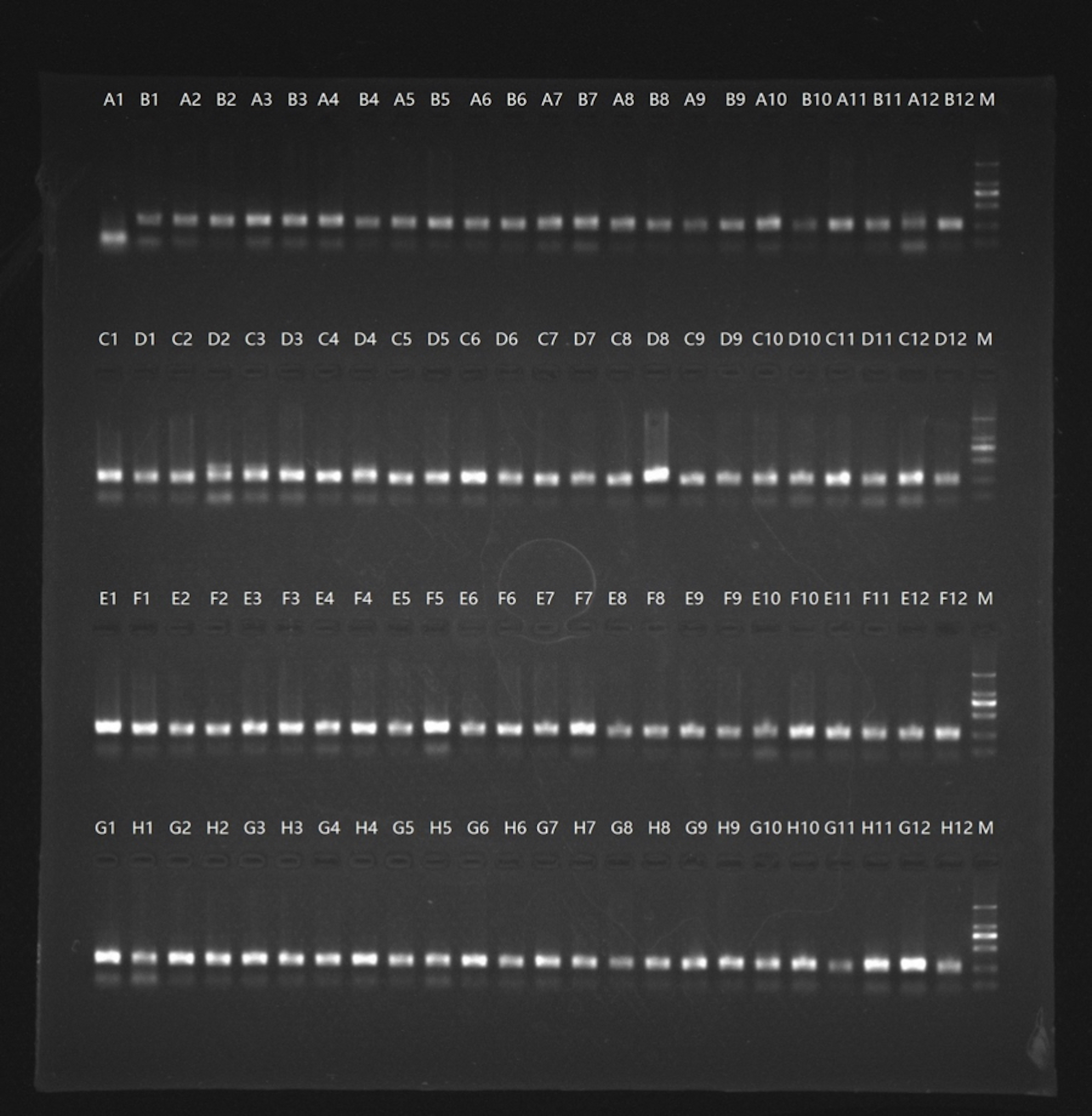

Supplement: Supplementary file 1 — Multiple PCR results of 94 cases of eligible samples for quality control. A1 for negative control, A2 for positive control, M for DL2000Marker, other symbols of B, C, D, E, F, G, H, and A3-A12 for all the 94 cases of samples. A-H were well positions, not sample IDs (jpg 4130 kb) [file 10528_2021_10039_MOESM1_ESM.jpg]

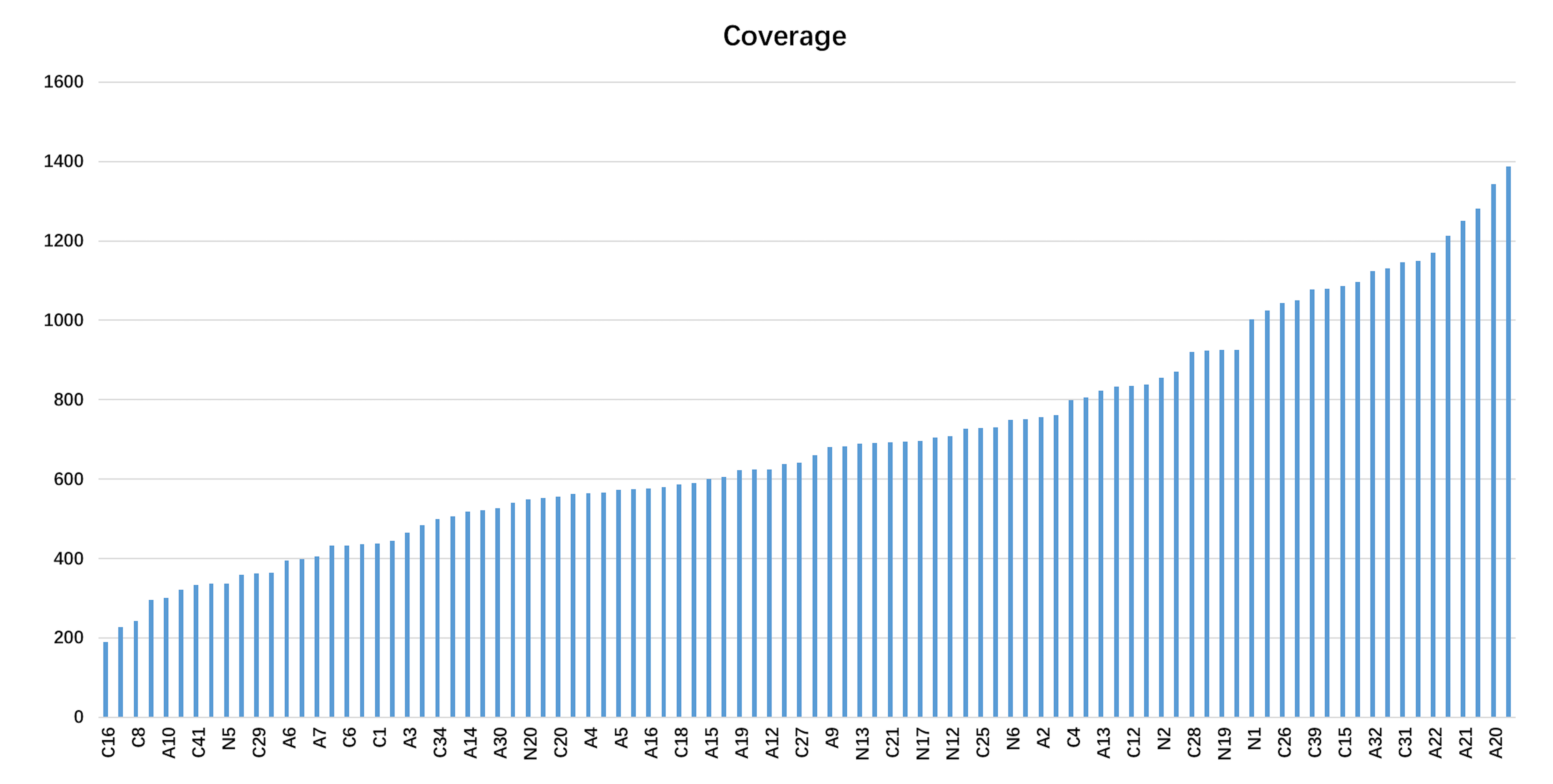

Supplement: Supplementary file 2 — Sequencing depth of all 94 samples for quality analysis. Ranged from 190X to 1536X, and the mean was 733X. The labels of samples were sample IDs (jpg 4485 kb) [file 10528_2021_10039_MOESM2_ESM.jpg]

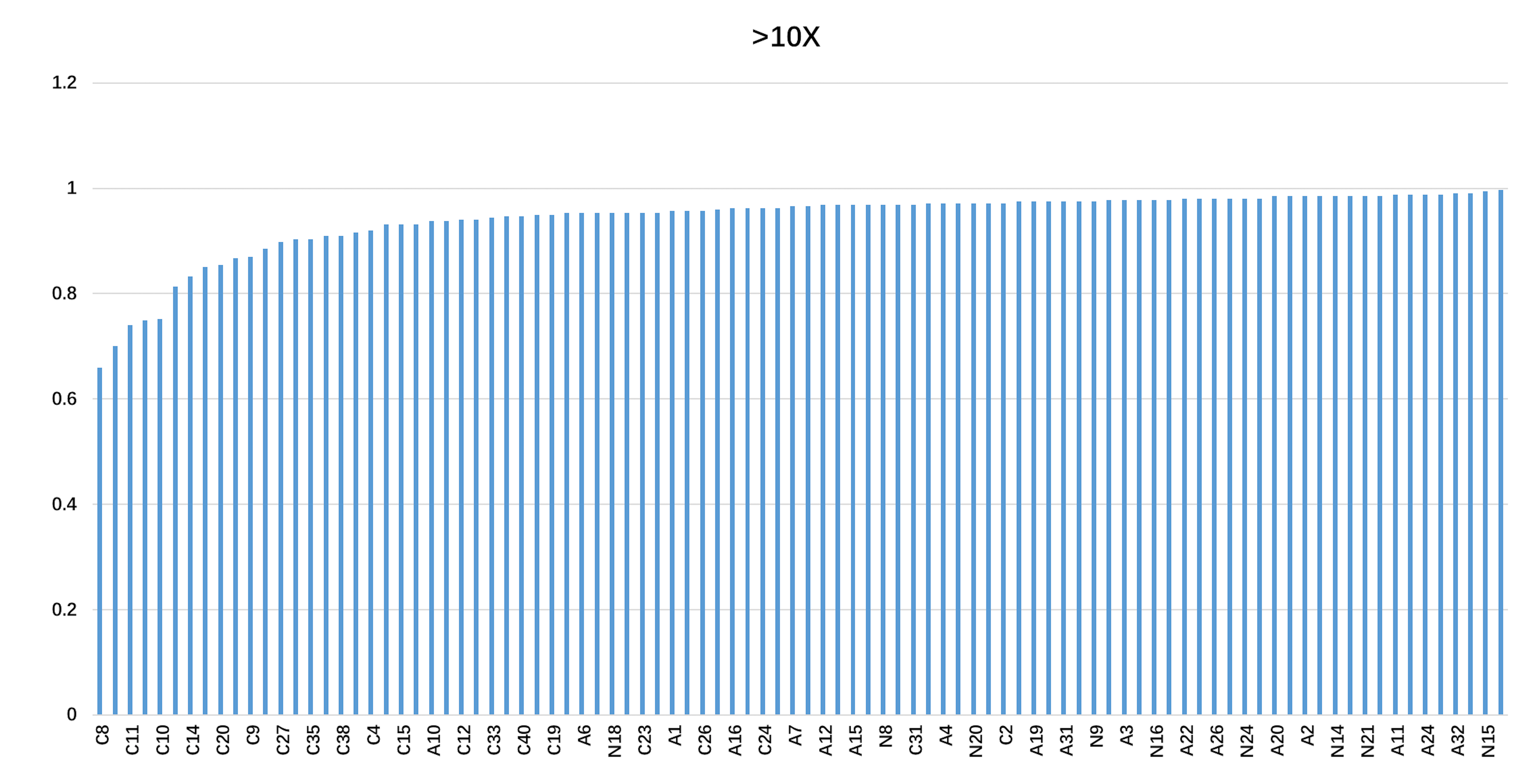

Supplement: Supplementary file 3 — Sequencing depth of target gene fragments was above 10X in 95.1% samples. The labels of samples were sample IDs (jpg 6262 kb) [file 10528_2021_10039_MOESM3_ESM.jpg]
