# Supplementary material for: Sum of High-Risk Gene Mutation (SHGM): A Novel Attempt to Assist Differential Diagnosis for Adrenocortical Carcinoma with Benign Adenoma, Based on Detection of Mutations of Nine Target Genes
Source: Biochem Genet. 2021 Feb 9;59(4):902–18. doi: 10.1007/s10528-021-10039-w (PMC8249247; doi:10.1007/s10528-021-10039-w)
Supplement: Supplementary file 4 — Electronic supplementary material 4 (DOCX 60 kb) [file 10528_2021_10039_MOESM4_ESM.docx]

Supplementary **Table 1.** **Main instruments and reagents**

| **Instruments** | **Origin** |
| --- | --- |
| Applied Biosystems 2720 Thermal Cycler | Applied Biosystems, USA |
| 3730xl Genetic Analyzer | Thermo Fisher Scientific，USA |
| Eppendorf 5810R Centrifuge | Eppendorf，Germany |
| NanoDrop 2000 Spectrophotomete | NanoDrop technologies, USA |
| Invitrogen Qbit Spectrophotometer | Invitrogen，USA |
| **MiSeq** Benchtop Sequencer | Illumina，USA |
| **Reagents** | **Origin** |
| HotstarTaq DNA Polymerase Kit | QIAGEN，Germany |
| Q5 DNA polymerase Kit | New England Biolabs，USA |
| GeneScan-500 Liz Size Standard | Thermo Fisher Scientific，USA |
| Agencourt AMPure XP-PCR Purification | Beckman，USA |
| **NextSeq**Reagent Kit v3 | Illumina，USA |
