# Supplementary material for: Sum of High-Risk Gene Mutation (SHGM): A Novel Attempt to Assist Differential Diagnosis for Adrenocortical Carcinoma with Benign Adenoma, Based on Detection of Mutations of Nine Target Genes
Source: Biochem Genet. 2021 Feb 9;59(4):902–18. doi: 10.1007/s10528-021-10039-w (PMC8249247; doi:10.1007/s10528-021-10039-w)
Supplement: Supplementary file 5 — Electronic supplementary material 5 (DOCX 90 kb) [file 10528_2021_10039_MOESM5_ESM.docx]

Supplementary Table 2.

| (a) DNA quality control | 1% agarose electrophoresis was used for DNA quality test. |
| --- | --- |
| (b) Primer design | Primers design of the 9 target genes was performed by FastTarget^TM^ primer design software (Genesky Biotechnologies Inc., Shanghai, China) |
| (c) Single site primer optimization | A 10μl mixture was prepared for each reaction and included 1x reaction buffer (TAKARA), 2 mmol Mg^2+^, 0.2 mmol dNTP, 0.2μmol of each primer, 1 U HotStarTaq polymerase (Takara) and 1 μl template DNA.  The cycling program was 95℃ for 2 min; 11 cycles of 95℃ for 20 s, 63℃-0.5℃ per cycle for 40 s, 72℃ for 1mins; 24 cycles of 95℃ for 20 s, 65℃ for 30 s, 72℃ for 1 min; 72℃ for 2 min. |
| (d) Multiple system optimization | According to the standard of 20 pairs of primers for each panel, the primers optimized in step (c) were mixed into multi PCR primer panel.  A 20μl mixture was prepared for each reaction and included 1x reaction buffer (TAKARA), 2 mmol Mg^2+^, 0.2 mmol dNTP, 0.1μmol of each primer, 1 U HotStarTaq polymerase (Takara) and 2 μl template DNA.  The cycling program was 95℃ for 2 min; 11 cycles of 94℃ for 20 s, 63℃-0.5℃ per cycle for 40 s, 72℃ for 1mins; 24 cycles of 94℃ for 20 s, 65℃ for 30 s, 72℃ for 1 min; 72℃ for 2 min. |
| (e) Multiplex PCR reaction of target fragments of samples | The optimized multiplex PCR primer panel was used to perform multiplex PCR reaction, and the sample genome was used as a template.  A 20μl mixture was prepared for each reaction and included 1x reaction buffer (TAKARA), 3 mmol Mg^2+^, 0.2 mmol dNTP, 0.1μmol of each primer, 1 U HotStarTaq polymerase (Takara) and 2 μl template DNA.  The cycling program was 95℃ for 2 min; 11 cycles of 94℃ for 20 s, 63℃-0.5℃ per cycle for 40 s, 72℃ for 1mins; 24 cycles of 94℃ for 20 s, 65℃ for 30 s, 72℃ for 1 min; 72℃ for 2 min. |
| (f) Add specific tag sequence | Using the primers with index sequence, specific tag sequences compatible with Illumina platform were introduced to the end of the library by PCR amplification.  A 20μl mixture was prepared for each reaction and included 1x reaction buffer (NEB Q5 TM), 0.3 mmol dNTP, 0.3μmol of F primer, 0.3μmol of index primer, 1 U Q5TM DNA polymerase (NEB) and 1 μl diluted template.  The cycling program was 98℃ for 30s; 11 cycles of 98℃ for 10 s, 65℃ for 30 s, 72℃ for 30 s; 72℃ for 5 min. |
| (g) Library quality control and sequencing | The final FastTarget library was obtained by mixing all the samples with the same amount of index PCR products and Gel Extraction. Fragment length distribution of the library was verified by Agilent 2100 Bioanalyzer.  After the accurate quantification of the library molar concentration, the high-throughput sequencing was carried out on Illumina Hiseq / Miseq Platform in the mode of 2 × 150 bp / 2 × 250 bp to obtain FastQ data. |
